# Effectiveness of the Facility for Elderly Surveillance System (FESSy) in Two Public Health Center Jurisdictions in Japan: Prospective Observational Study

**DOI:** 10.2196/58509

**Published:** 2025-01-10

**Authors:** Junko Kurita, Motomi Hori, Sumiyo Yamaguchi, Aiko Ogiwara, Yurina Saito, Minako Sugiyama, Asami Sunadori, Tomoko Hayashi, Akane Hara, Yukari Kawana, Youichi Itoi, Tamie Sugawara, Yoshiyuki Sugishita, Fujiko Irie, Naomi Sakurai

**Affiliations:** 1 Department of Nursing, Faculty of Sports & Health Science, Daito Bunka University, Higashimatsuyama-shi, Japan; 2 Graduate School of Health Sciences (Nursing), Ibaraki Prefectural University of Health Sciences, Ami-machi, Japan; 3 Sumida City Public Health Center, Sumida City, Japan; 4 Tsuchiura Public Health Center, Tsuchiura City, Japan; 5 National Institute of Infectious Diseases, Shinjuku City, Japan; 6 Center for Medical Sciences, Ibaraki Prefectural University of Health Sciences, Ami-machi, Japan

**Keywords:** early detection, facility for older people, outbreak, public health center, syndromic surveillance, Japan, older adults

## Abstract

**Background:**

Residents of facilities for older people are vulnerable to COVID-19 outbreaks. Nevertheless, timely recognition of outbreaks at facilities for older people at public health centers has been impossible in Japan since May 8, 2023, when the Japanese government discontinued aggressive countermeasures against COVID-19 because of the waning severity of the dominant Omicron strain. The Facility for Elderly Surveillance System (FESSy) has been developed to improve information collection.

**Objective:**

This study examined FESSy experiences and effectiveness in two public health center jurisdictions in Japan.

**Methods:**

This study assessed the use by public health centers of the detection mode of an automated AI detection system (ie, FESSy AI), as well as manual detection by the public health centers’ staff (ie, FESSy staff) and direct reporting by facilities to the public health centers. We considered the following aspects: (1) diagnoses or symptoms, (2) numbers of patients as of their detection date, and (3) ultimate numbers of patients involved in incidents. Subsequently, effectiveness was assessed and compared based on detection modes. The study lasted from June 1, 2023, through January 2024.

**Results:**

In both areas, this study examined 31 facilities at which 87 incidents were detected. FESSy (AI or staff) detected significantly fewer patients than non-FESSy methods, that is, direct reporting to the public health center of the detection date and ultimate number of patients.

**Conclusions:**

FESSy was superior to direct reporting from facilities for the number of patients as of the detection date and for the ultimate outbreak size.

## Introduction

**Figure 1.**
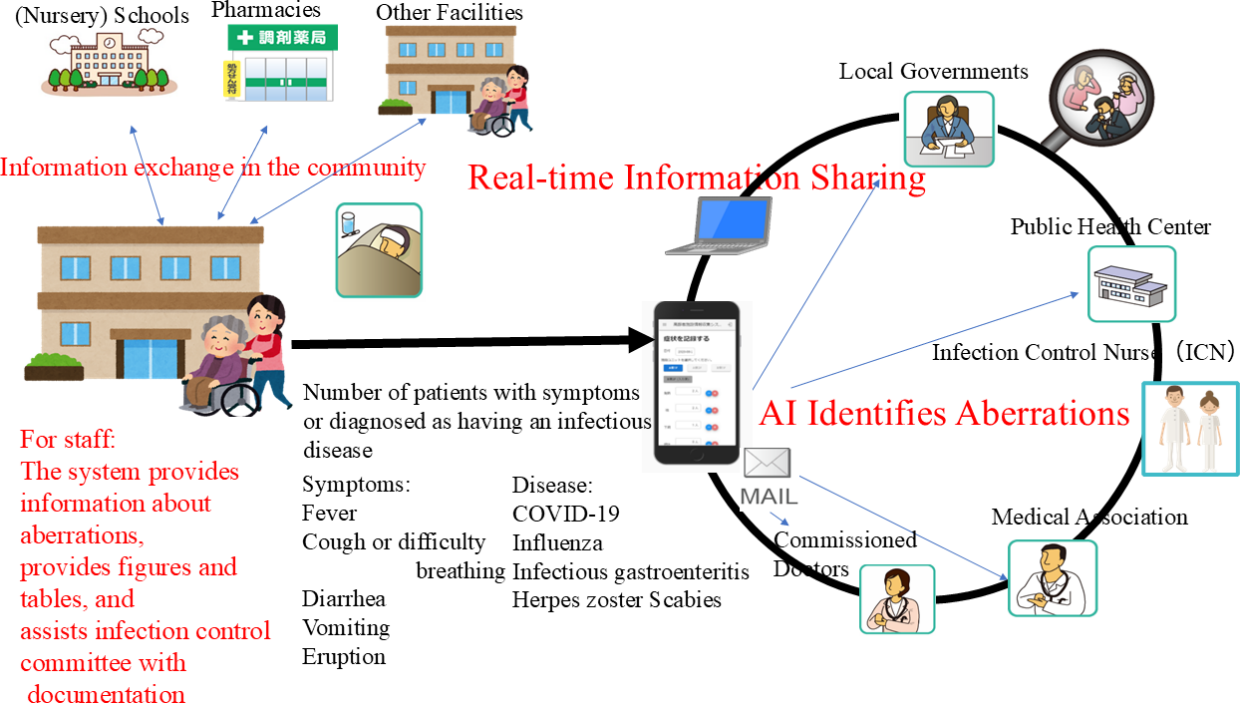
Concept of the Facility for Elderly Surveillance System (FESSy). Facilities enter data (shown on the left) on the number of patients with certain symptoms or infectious diseases; this is shared with stakeholders that include public health centers, local governments, infection control nurses, commissioned doctors, and the medical association (shown in the circle on the right). Moreover, the FESSy AI monitors the entered data to identify aberrations through comparison with past aberration data and patterns, as judged by the public health center staff or developers. When the AI finds an aberration, it sends an email to the stakeholders and FESSy facility correspondents.

Residents at facilities for older people have remained remarkably vulnerable to COVID-19 outbreaks [1]. Infection control at such facilities is expected to be the most important method to reduce the disease burden attributable to COVID-19 [2]. However, on May 8, 2023, infection control policies for COVID-19 in Japan were relaxed. For that reason, public health centers have become unable to recognize outbreak situations at facilities for older people unless the outbreak grows to include more than 10 cases [3]. To fill this information gap, the Facility for Elderly Surveillance System (FESSy) has been used [4] as a mode of syndromic surveillance that monitors symptoms and infectious diseases among facility residents. This web data exchange system transmits data entered from a smartphone or PC. Each facility inputs data on the number of residents or staff members with certain symptoms or who have been diagnosed as having infectious diseases in units where residents cohabitate and unit staff members manage them. Figure 1 presents the system concept. The targeted symptoms include fever, cough or difficulty breathing, vomiting, diarrhea, and eruption. The diagnosed infectious diseases include COVID-19, influenza, infectious gastroenteritis, herpes zoster, and scabies. Data aberrations can be detected by the unit at each facility using artificial intelligence (AI). If an aberration is detected, this information is then delivered to the commissioned doctors, public health centers, medical associations, infection control nurses in the community, and local governments, who are allowed access to data entered at the facility, as shown on the right-hand side of Figure 1.

Development of FESSy was begun in 2008 by a research group headed by Dr Yasushi Ohkusa of the National Institute of Infectious Diseases, with trials conducted at a few facilities. However, use of FESSy and the related research were then suspended for an extended period. Recently, an author of this study (JK) developed a smartphone version of FESSy and has renewed use of the system and related research. Currently, its copyright is held by a nonprofit organization that took over the project from the original research group and a private company (Milabo Co Ltd). It has been functioning in Ibaraki prefecture and Sumida City in Tokyo as part of research group activities headed by author NS.

Two public health centers operating FESSy in Sumida City in Tokyo and Tsuchiura in Ibaraki prefecture cooperated in this study. Sumida City, which is located in the center of metropolitan Tokyo, has approximately 280,000 residents. The Tsuchiura Public Health Center jurisdiction comprises 3 cities with a total population of 250,000. In Japan, most medical services are financed by national public health insurance. The same policies for older persons apply in all areas in Japan. In this sense, Sumida City and Tsuchiura present similar situations in terms of health care and welfare. Sumida City is a somewhat more urban area when compared with Tsuchiura. FESSy was activated in March 2023 in Sumida City. It was activated in September 2023 in Tsuchiura based on a request from the Ibaraki Prefectural University of Health Sciences. The Tsuchiura Public Health Center jurisdiction includes 31 long-term care facilities (special nursing homes for older people). There are 10 long-term care facilities in Sumida City.

Soon, FESSy is expected to be activated nationwide, where it can contribute to timely detection and rapid infection control at facilities. This study was conducted to summarize experiences of FESSy and to evaluate its effectiveness.

A system similar to FESSy has reportedly been operational in the Netherlands since April 2017 [5]. The Meldpunt voor Uitbraken van Infectieziekten en Bijzonder Resistente Micro-organismen (MUIZ) is a system for recording outbreaks at facilities and for the timely sharing of information, although it does not clearly define outbreaks for diseases other than COVID-19. All COVID-19 cases since March 2020 should have been recorded through the system, as it operated during the pandemic. However, it remains unknown how it has operated after the pandemic. It is noteworthy that the system does not function until an outbreak emerges. Therefore, unlike FESSy, MUIZ might not be suitable for early intervention and outbreak prevention for diseases other than COVID-19.

In Australia, the Aged Care National Antimicrobial Prescribing Survey and Victorian Healthcare Associated Infection Surveillance System operate at some facilities [6]. However, these facilities join voluntarily; like FESSy, participation is not enforced by law. The Australian system is aimed at saving time used for data entry, which must also be considered in Japan [7].

## Methods

### Overview

This study was conducted at the Sumida City Public Health Center in Tokyo and at the Tsuchiura Public Health Center in Ibaraki prefecture. Study participants were limited to patients at facilities for older people in the jurisdictions of the 2 public health centers, as we particularly wanted to study long-term care facilities, specifically, special nursing homes for older people. The study period was from June 2023 to the end of January 2024. If a facility joined FESSy during the study period, data that the facility had accumulated before joining FESSy were ignored.

In each unit of the facility, FESSy detects aberrations using AI, based on the “C1-MILD” method of the Early Aberration Reporting System (EARS) [8], which includes symptoms and diagnoses. In EARS, C1-MILD defines a data aberration as being 3 times the standard deviation during the prior 7 days and higher than average in the same period. If the AI finds an aberration, then emails are sent automatically to facility managers or staff, public health centers, local governments, commissioned doctors, infection control nurses in the community, and medical associations. Hereafter, we refer to this AI detection mode in FESSy as “FESSy AI.” Based on email notifications from FESSy AI, a public health center can activate a public health intervention at a facility. Of course, public health center staff can always monitor the situation at facilities using FESSy, even if they have not yet received an alert email from FESSy AI. Sometimes a very early-stage outbreak is identifiable by staff, facilitating a rapid public health response. We refer to this detection mode as “FESSy staff.” Aside from FESSy AI and FESSy staff, a public health center sometimes receives a report from a facility directly, rather than via FESSy. We refer to that as a “direct report.”

First, public health center operations can be generalized into 3 dimensions for all detection modes (FESSy AI, FESSy staff, and direct report): (1) diseases or symptoms, (2) number of patients as of the detection date, and (3) ultimate number of patients involved in the incident. Regarding (1), examination of diseases or symptoms can engender multiple answers.

We tested the overall FESSy effectiveness (sum of results for FESSy AI and FESSy staff) compared to the non-FESSy method, which can only operate based on direct reports from facilities. Moreover, we sought to ascertain, by testing, which was the more effective of the 2 FESSy communication modes: FESSy AI or FESSy staff. The effectiveness of each detection mode was measured as the number of patients as of the detection date or as of the end of the outbreak. The Wilcoxon rank-sum test was used to compare FESSy versus non-FESSy methods, as well as FESSy AI versus FESSy staff. We adopted 5% as the significance level and used software (Stata SE version 17.0; Stata Corp) for all statistical analyses.

### Ethical Considerations

This study was approved by the Ethics Committee of Ibaraki Prefectural University of Health Sciences on August 21, 2023 (1103); the applicant was the corresponding author (JK). We obtained written informed consent from the two public health centers.

## Results

**Figure 2.**
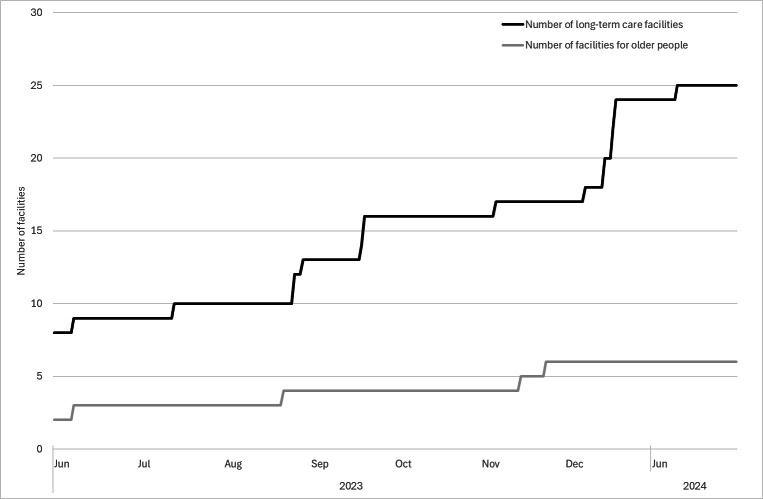
Number of facilities joining the Facility for Elderly Surveillance System (FESSy) in the Sumida City Public Health Center and the Tsuchiura Public Health Center from June 1, 2023, to January 31, 2024. The black line represents the number of long-term care facilities joining FESSy. The gray line represents the number of facilities for older people joining FESSy, other than long-term care facilities. In the Tsuchiura Public Health Center jurisdiction, FESSy started operation on September 1, 2023. Finally, 14 facilities had joined FESSy in the Sumida Public Health Center jurisdiction and 17 facilities had joined FESSy in the Tsuchiura Public Health Center jurisdiction as of the end of January 2024.

As of the end of January 2024, 17 facilities had joined FESSy that were under the jurisdiction of the Tsuchiura Public Health Center: 16 were long-term care facilities (special nursing homes for older people) and 1 was a geriatric health services facility. In Sumida City, 14 facilities joined FESSy, of which 9 were long-term care facilities; others included geriatric health services facilities, low-cost homes for older people, and fee-based facilities. The proportion of long-term care facilities that joined FESSy was 53% (16/30 facilities) in the Tsuchiura Public Health Center and approximately 90% (9/10 facilities) in the Sumida City Public Health Center as of the end of January 2024. The increases in the number of facilities joining in the two areas are shown in Figure 2.

**Figure 3.**
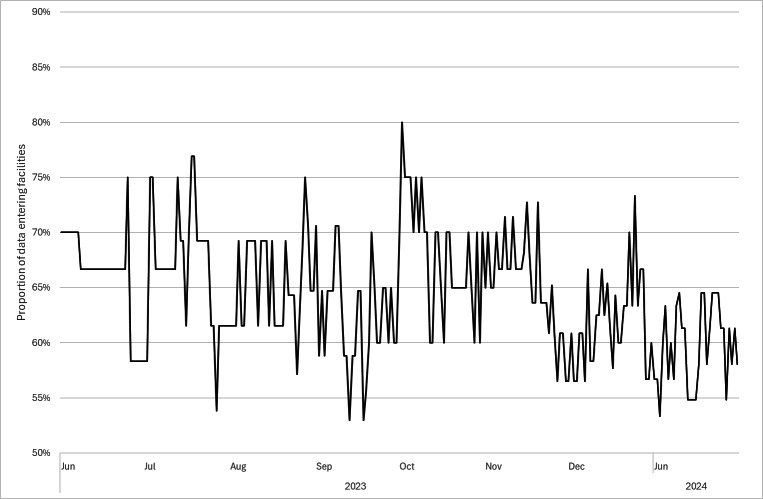
Proportion of facilities that entered data into the Facility for Elderly Surveillance System (FESSy) in the jurisdictions of the Sumida City Public Health Center and Tsuchiura Public Health Center from June 1, 2023, to January 31, 2024. Proportions for data entry were defined as the number of facilities that entered data in a day divided by the number of facilities that had joined FESSy as of the dates shown in Figure 2. A facility was defined as having entered data if it had entered information on symptoms, including an absence of patients with a certain symptom. Twelve facilities entered data every day, while another 10%-30% of facilities entered data but did not do so every day.

Figure 3 presents the proportion of data entry at FESSy facilities, which fluctuated between 50% and 80%. However, the average was almost 65%. In fact, some facilities that had joined never entered data into the FESSy system. In addition, facilities that immediately started to enter data into FESSy continued to enter data every day.

**Figure 4.**
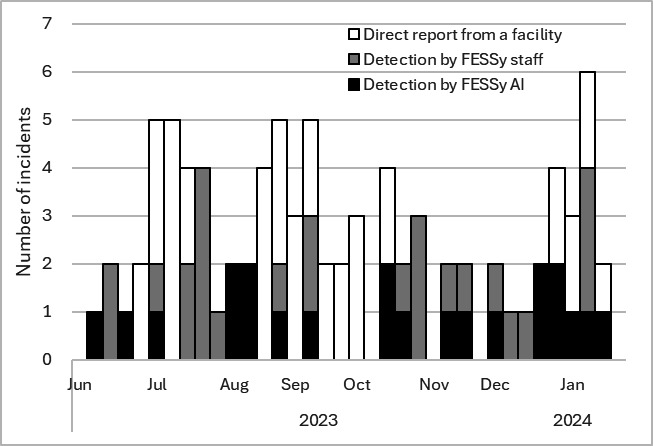
Number of incidents per week by detection mode for the Facility for Elderly Surveillance System (FESSy) at Sumida City Public Health Center and Tsuchiura Public Health Center from June 1, 2023, to January 31, 2024.

During the study period, 87 incidents were detected in both jurisdictions. Figure 4 presents the number of incidents by week and by detection mode. More than half of all incidents were detected through FESSy AI (n=21 incidents) and FESSy staff (n=26 incidents). Aside from those, 40 incidents were reported directly to the public health centers.

**Figure 5.**
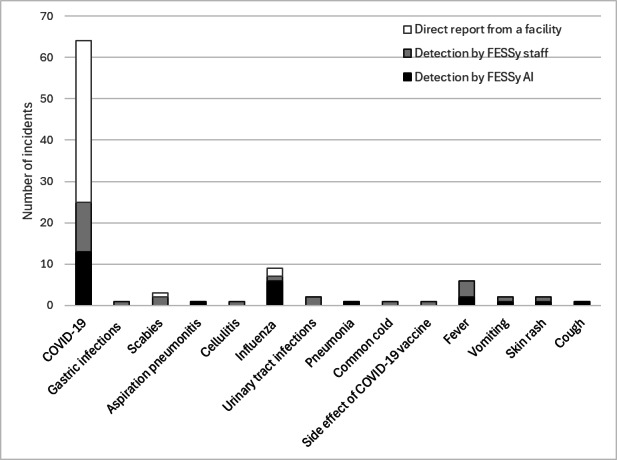
Numbers of incidents by disease or symptom and by detection and notification modes of the Facility for Elderly Surveillance System (FESSy) in Sumida City Public Health Center and Tsuchiura Public Health Center from June 1, 2023, to January 31, 2024. Terms from “COVID-19” through “side effect of COVID-19 vaccine” are diseases. Terms from “fever” through “cough” are symptoms. There were a total of 95 incidents with disease or symptoms (some incidents were caused by more than 1 disease or symptom).

Figure 5 shows the distribution of diseases and symptoms by detection mode. The incidents caused by COVID-19 were the most numerous at 63. The next most frequent incidents were caused by influenza, with 8 incidents. Incidents detected by direct reports were limited mainly to COVID-19, with 2 incidents of influenza and 1 incident of scabies. Conversely, FESSy AI and FESSy staff found aberrations for diseases other than COVID-19, including symptoms such as fever, vomiting, and rash.

**Figure 6.**
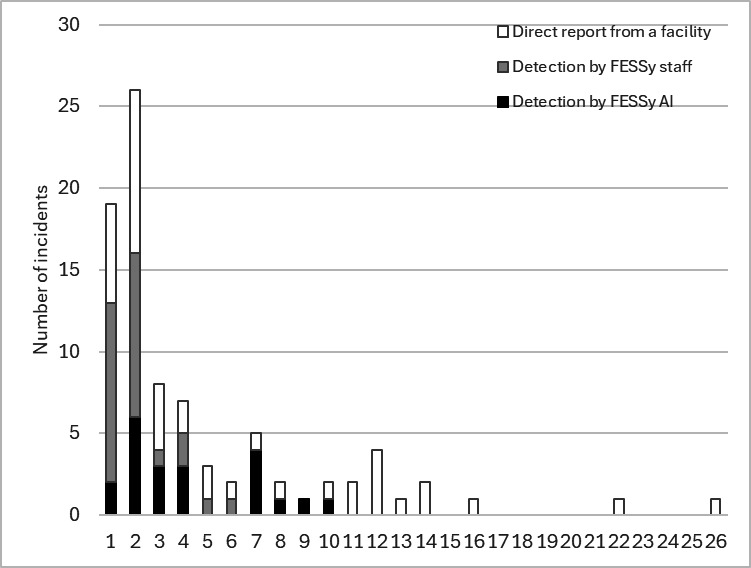
Numbers of incidents by the number of patients as of the detection date and by detection mode of the Facility for Elderly Surveillance System (FESSy) in Sumida City Public Health Center and Tsuchiura Public Health Center from June 1, 2023, to January 31, 2024. One detected incident did not include the number of patients detected. The total number of incidents was 87.

Figure 6 presents the distribution of the number of patients as of the detection date by detection mode. One reported incident did not include the number of patients. The mode for number of patients was 2. The largest number of patients was 26, which was attributable to a COVID-19 outbreak. Incidents with more than 11 patients were limited to detection by direct report. By detection mode, the average number of patients detected by FESSy AI was 4.29 (SD 2.80) for 21 incidents. For FESSy staff, the average number was 1.83 (SD 1.09) for 26 incidents. For direct reports, it was 6.55 (SD 6.18) for 40 incidents. The estimated *P* value of a rank-sum test comparison between FESSy and non-FESSy methods was .004. The *P* value was <.001 for a comparison of FESSy AI and FESSy staff.

**Figure 7.**
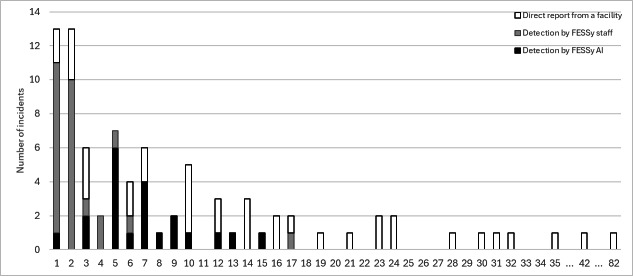
Number of incidents by ultimate number of patients and by detection mode of the Facility for Elderly Surveillance System (FESSy) in Sumida City and Tsuchiura from June 1, 2023, to January 31, 2024. The total number of incidents was 84. Three incidents were ongoing at the end of the study period; the ultimate number of patients was thus not available.

Figure 7 shows a distribution of the ultimate number of patients involved in incidents by detection mode. Three incidents did not include this information because of the outbreak was ongoing at the end of the study period. The mode for number of patients was 1. The largest number of patients was 82, which was attributable to a COVID-19 outbreak. Incidents with more than 19 patients were limited to detection by direct report. By detection mode, the average number of patients detected by FESSy AI was 7.00(SD 3.60) for 21 incidents. For FESSy staff, the average number was 2.52 (SD 3.25) for 26 incidents. For direct report, it was 16.5 (SD 15.4 for 37 incidents. The estimated *P* value of a rank-sum test comparison of FESSy and non-FESSy methods was <.001. The P value was .001 for a comparison of FESSy AI and FESSy staff.

## Discussion

### Principal Findings

Findings from statistical analysis indicate that FESSy detected outbreaks earlier than non-FESSy methods (eg, direct reporting). Notably, FESSy staff could detect an outbreak earlier than FESSy AI. Similarly, the ultimate number of patients was smaller for incidents detected by FESSy than by non-FESSy methods. Figure 5 shows that FESSy AI and FESSy staff detected aberrations for diseases other than COVID-19 and for certain symptoms. This finding suggests the usefulness of FESSy, for which detection is not limited to a particular disease, in contrast to MUIZ in the Netherlands, which is limited to COVID-19.

Comparison of the FESSy detection modes, FESSy AI and FESSy staff, showed that the latter was more timely than the former. FESSy staff sometimes detected an aberration with only 1 person. In this situation, AI usually inferred a nonaberration. In fact, the C1-MILD method was too sensitive: it produced many false positive results because it referred only to the prior 7 days. The use of AI reduced false positives by incorporating information from a longer period or that was obtained with other methods for aberration inference, including FESSy staff. Particularly, Figure 6 shows that, with few exceptions, AI did not recognize the initiation of an outbreak if the number of patients was merely 1. Therefore, a FESSy AI notification might be sent later than a FESSy staff notification. Regarding outcomes, the number of patients at the time of detection might be appropriate to evaluate timeliness. However, that measure does not include information about final outcomes, such as the outbreak size or whether mortality cases are included. In other words, the number of patients as of the time of detection can reflect timeliness or sensitivity, but might not reflect specificity for an aberration.

Evaluation of specificity not only for FESSy but also for syndromic surveillance in general was quite difficult because, unlike laboratory tests, no gold standard exists for surveillance. In the case of FESSy, specificity was defined as not detecting aberrations that would not develop into large outbreaks. Thus, to determine specificity for FESSy, one would have to know the proportion of aberrations found by FESSy that would not develop into a large outbreak if countermeasures were not taken. However, in the real world, FESSy immediately found aberrations, after which countermeasures at the facility or public health center were activated to reduce the probability of these aberrations developing into large outbreaks. Because FESSy was so effective, the high probability of an outbreak was removed. In other words, we could not observe a situation at a FESSy-affiliated facility in which FESSy detected no aberration. Moreover, at non-FESSy facilities, it was difficult to find aberrations that occurred with the same timing according to which FESSy usually detects an aberration. Public health centers sometimes recognize a situation at such facilities after an outbreak has already expanded. Because of these difficulties and paradoxical comparisons, we anticipate that evaluation of specificity will be a future challenge for the evaluation of FESSy.

On May 8, 2023, the government changed the criterion for reporting COVID-19 infections at facilities to a public health center: instead of reporting all cases, reporting was relaxed to the reporting of only clusters involving more than 10 cases. However, the modes for the number of patients detected by direct reports in this study were 2 and 1 at the two centers. This finding might indicate that the earlier criterion for reporting to a public health center (all cases) continued in facilities even after May 8, 2023, depending on spontaneous decisions at facilities, even though public health centers were never required to report all cases after May 8, 2023.

Before FESSy initiation in both areas, we were concerned about burdens imposed by data entry at the facilities. Nevertheless, no complaints have been made by facility staff members about data entry burdens. The data were entered regularly every day. To reduce the data entry burden at facilities joining FESSy, we developed a method for automatic data transfer from electronic care records at facilities [7], as suggested by the results of a study in Australia [6]. However, because the share of electronic care records including symptoms and diagnoses that could be transferred automatically as data was not very high, use of the method has been limited to a small number of facilities. We expect that other ideas to reduce the data entry burden will be necessary.

Moreover, information from FESSy has been shared with public health centers. That information has contributed to the detection of outbreaks of COVID-19 and other infectious diseases in a timely manner. Developing situations are followed up without additional effort. Infection control nurses in communities or at affiliated hospitals and public health nurses at public health centers in particular highly regarded its effectiveness at controlling outbreaks.

In Tsuchiura, the proportion of facilities joining FESSy remained at 51% (n=16 facilities), much lower than the corresponding proportion in Sumida City of 90% (n=9 facilities). However, the number of facilities joining FESSy was larger in Tsuchiura than in Sumida City. Therefore, this difference might reflect only the number of facilities covered by the public health center, irrespective of those participating in FESSy. We consider that there is no fundamental difference between them. Moreover, the program was introduced in Tsuchiura 3 months later than in Sumida City. Thus, we expect that the proportion of participating facilities in Tsuchiura will increase in the coming months. We will examine whether there are any difficulties associated with FESSy in Tsuchiura through surveys of facilities. If some particular difficulty is found, then a challenge for future research will be to solve it.

Study periods were insufficiently long: 8 months for Sumida City and 5 months for Tsuchiura. However, we found that FESSY had some degree of effectiveness. Earlier publication of this fact might have contributed to a more rapid spread of FESSy participation, even though we expect that ongoing verification of the robustness of our results will be necessary.

Finally, we used an AI system to detect aberrations. In FESSy, AI support for public health center staff awareness of situations can contribute to a reduced burden of monitoring the system. When the AI detects an aberration, the decision-making about what actions to take as countermeasures and how to respond to the outbreak are made by public health center staff. Therefore, the AI itself can take no action after detecting an aberration other than sending email messages to an established list of recipients. Because the AI in our system only monitors data in FESSy, including the number of patients with certain symptoms and diagnoses, the AI never refers to the general internet outside of FESSy. Therefore, no ethical issues arose in relation to using AI in FESSy [9]. Moreover, the AI in FESSy has been learning about the criteria used for aberration detection by humans, public health center staff, facilities, and others to improve its own criteria. Therefore, the AI criteria will not deviate from the human criteria. If the number of participating facilities increases rapidly, then the AI criteria might converge to the human criteria in the near future.

### Limitations

First, this study was an evaluation conducted at only 2 public health centers. The results might depend on the characteristics of the facilities or the public health centers. The findings’ robustness must be verified through similar evaluations of FESSy conducted in other cities with other public health centers.

Second, the sample size of this study was too small for the AI to study and learn aberration patterns, especially those found by the FESSy staff. A longer period and a wider area can be expected to alleviate this limitation. In this sense, the results obtained from this study must be regarded as tentative, not final.

Third, data entry at participating FESSy facilities was based on free decisions made by the facility management. Therefore, facility participation in FESSy and data entry in FESSy might have been biased, reflecting especially strong concerns about infectious disease. That bias might have led to overestimation of FESSy’s effectiveness. This is particularly the case in Tsuchiura because the proportion of facilities participating in FESSy was much smaller than in Sumida City, so the potential bias might be stronger. We cannot randomly assign participation in FESSy or data entry in FESSy, but an average treatment effect model might be able to resolve these issues. That is left as a challenge for a future study.

### Conclusion

Our results show that FESSy was superior to direct reporting from facilities, which was the only method available in areas where FESSy had not been activated, not only for the number of patients as of the detection date but also the ultimate outbreak size. As a useful tool for public health centers, FESSy clearly improved the public health situations in both areas it was used.

Recently, special cities in Tokyo, including Sumida City, have been obligated to construct infection prevention plans based on a Japanese law, the Act on the Prevention of Infectious Diseases and Medical Care for Patients with Infectious Diseases. If FESSy were incorporated into the plan, it would support stable infection control measures before the emergence of outbreaks.

## Abbreviations

EARS: Early Aberration Reporting System
MUIZ: Meldpunt voor Uitbraken van Infectieziekten en Bijzonder Resistente Micro-organismen

## References

[1] Shimizu K, Maeda H, Sando E (2022). Epidemiology of SARS-CoV-2 infection in nursing facilities and the impact of their clusters in a Japanese core city. J Infect Chemother.

[2] Vijh R, Ng CH, Shirmaleki M, Bharmal A (2022). Factors associated with transmission of COVID-19 in long-term care facility outbreaks. J Hosp Infect.

[3] The reporting of infectious disease outbreaks in social welfare facilities (revised on April 28, 2023). Ministry of Health, Labour and Welfare.

[4] Kurita J, Sugawara T, Ohkusa Y, Sakurai N (2023). Experiment of surveillance for long-term care facilities for elderly people. Bull of Daito Bunka Univ.

[5] Meima A, Whelan J, Dijks J, van der Hagen N, van Duuren M, Tjon-A-Tsien A (2023). Introducing a novel “real-time” outbreak alert and notification system to monitor SARS-CoV-2 outbreaks and case fatality in elderly care facilities, the Netherlands, 2020-2022. J Public Health Res.

[6] Watson E, Dowson L, Dunt D (2023). Identifying barriers and enablers to participation in infection surveillance in Australian residential aged care facilities. BMC Public Health.

[7] Kurita J, Sakurai N (2024). An examination for automatic upload function in elderly facility surveillance system from electronical care record [in Japanese]. Jpn J Med Inform.

[8] Hutwagner L, Thompson W, Seeman GM, Treadwell T (2003). The bioterrorism preparedness and response Early Aberration Reporting System (EARS). J Urban Health.

[9] Ethics of artificial intelligence. United Nations Educational, Scientific and Cultural Organization (UNESCO).

